# Effects of replacing concentrates with cassava products on feed intake, digestibility, and rumen fermentation in Thai native cattle

**DOI:** 10.5455/javar.2025.l977

**Published:** 2025-12-25

**Authors:** Sophany Morm, Areerat Lunpha, Ruangyote Pilajun, Anusorn Cherdthong

**Affiliations:** 1 Department of Animal Science, Faculty of Agriculture, Ubon Ratchathani University, Ubon Ratchathani, Thailand; 2 Department of Animal Science, Faculty of Agriculture and Food Processing, National University of Battambang, Battambang, Cambodia; 3 Department of Animal Science, Faculty of Agriculture, Khon Kaen University, Khon Kaen, Thailand

**Keywords:** Cassava tops, Cassava pulp, Dried cassava tops, Fermented cassava pulp

## Abstract

**Objective:**

The study investigated the effect of replacing concentrate with dried cassava tops and fermented cassava pulp (CtFCp) on feed efficiency, nutrient intake, and rumen fermentation in Thai native cattle.

**Materials and Methods:**

A completely randomized design with three dietary treatments was conducted with female Thai native cattle, initially weighing approximately 97 kg. The dietary treatments consisted of 100% concentrate (Control), 67% concentrate mixed with 33% dried CtFCp-33, and 33% concentrate mixed with 67% dried CtFCp-67, all based on dry matter (DM).

**Results:**

DM intake and digestibility were lower (*p* < 0.05) in the CtFCp-67 treatment than in other treatments. In addition, crude protein digestibility increased (*p* < 0.001) in the CtFCp diets. At 4 h post-feeding, concentrations of acetate and propionate were significantly decreased in the CtFCp-67 treatment, while total volatile fatty acids and butyrate were also significantly decreased (*p* < 0.05). Estimated methane emissions were lower in CtFCp-67 than CtFCp-33 (*p* < 0.05).

**Conclusion:**

CtFCp can replace up to 33% of concentrate in Thai native cattle diets without affecting intake or digestibility. Further studies should assess the effects of lactating cows.

## Introduction

Cassava is a valuable alternative food source for humans and animals, and it can promote economic development in developing African and Asian societies [1]. Cassava tops, which are combined green stems from leaf to buds, are approximately 20–30 cm in length and include leaves and petioles, as described by Morm et al. [2]. Dried cassava leaves contained 20%–30% crude protein (CP), and fresh cassava foliage was reported at 21.87%–24.8% [3], 16.07% [1], and 40%–44% [4]. 20.76%–21.55% of CP was contained in cassava leaves when fermented with molasses [3]. Using cassava foliage may enhance an energy-dense diet and improve the nutritional value of animal feed. To enhance cattle productivity [5], various feed additives have been used, including yeast-derived products like *Saccharomyces cerevisiae*, which are particularly beneficial for rumen function and overall animal health. The cassava leaves showed a high cyanide concentration, which was not safe for human consumption without proper processing [6,7]. Boukhers et al. [7] elucidated that it serves not only for consumption but also has natural medical purposes, with potent bioactivity interests for human health, and prevents inflammation.

Cassava root (CR) approximately contained 30% of cassava by-product [8]. In addition, cassava pulp (CSP) contains little protein but is rich in starch, serving as a primary source of energy [9]. This aspect makes it particularly valuable in animal feed formulations, where energy density is crucial for growth and production. Furthermore, the use of CSP can contribute to more sustainable agricultural practices by minimizing waste in cassava processing. To increase nutritional value and enhance feedstuff quality, fermented cassava pulp (FCSP) with molasses and urea was suggested [10]. Feed costs may be reduced due to increased CP efficiency when cassava tops are fermented with live yeast. Additionally, CSP improves nutrient digestibility, feed intake, methane production, and reproductive performance [2,9]. Phesatcha et al. [11] have shown that yeast is frequently utilized in the gastrointestinal systems of beef cattle as both a probiotic and a prebiotic. Yeasts are capable of removing oxygen in the rumen and may help manage the potential for hydrogen sulfide by competing with lactic acid bacteria, thereby minimizing the risk of acidosis [12]. Moreover, feeding CSP to Thai native cattle did not affect energy intake, heat production, and energy utilization efficiency. The total digestibility of nutrients, digestible energy, and metabolizable energy (ME) of CSP was reported as 74.4%, 12.9 MJ/kg, and 11.3 MJ/kg on a dry matter (DM) basis, respectively, according to Kabsuk et al. [13]. In addition, CSP enhances microbial protein (MCP) synthesis, nutrient digestibility, rumen ecology, and nitrogen utilization efficiency when supplemented at 100% for yearling crossbred beef cattle [14]. Furthermore, CSP increases the level of acetic acid, lactic acid, propionic acid, and CP, while fermented urea, molasses, and *Lactobacillus casei* TH14 were used for 21 days [15]. Alternatively, by replacing soybean meals with yeast-FCSP, the digestibility of nutrients in organic matter (OM) and CP increased [16].

Total mixed ratio and CSP fermentation enhanced digestibility and nutrient availability in lambs, as measured by acid detergent fiber (ADF), neutral detergent fiber (NDF), DM, OM, and CP [17], corroborating earlier findings [2] and the current study. Jiang et al. [8] noted that high concentrations boosted nutrient digestibility and intake in cattle-yak. Providing 70% concentrate in goats’ diets can enhance feed intake and OM digestibility, but does not affect fat yield nor increase total fatty acid concentration. Thus, the concentrate is a crucial factor in reducing ruminal ammonia concentrations while enhancing the utilization of ammonia for milk protein synthesis in dairy cows when provided in high concentrations, as in the diet [18]. Thus, rumen fermentation and rumen microbes increased in terms of nutritional enhancement when concentrate was included in dairy cows’ diets [11]. Moreover, cassava residue has been shown to affect production when used as concentrates for Holstein cows by Zheng et al. [19].

Our synthesis indicates that there is currently no evidence to suggest that cassava tops and fermented cassava pulp (CtFCp), combined with *S*. *cerevisiae*, enhance nutritional value, digestibility, feed intake, nutrient utilization, rumen fermentation, or blood metabolites when incorporated into concentrate in previous studies. Thus, the main objective was to investigate the effects of replacing dried CtFCp on feed efficiency, nutrient intake, rumen fermentation, and rumen microbial populations in Thai native cattle.

## Materials and Methods

### Ethical approval

The Thai native cattle used in the experiment followed the guidelines of Animal Care Ethics as outlined by the National Research Council of Ubon Ratchathani University (UBU), Thailand, No. HMESI 0604/2565.

### Preparation of cassava fermented product

The fresh cassava tops (*Manihot esculenta* Kasetsart 50), which consisted of cassava tops combined with green stems approximately 20–30 cm in length from leaf buds, including leaves and petioles, were purchased from a local farmer in Ban Hare (Tambon Kham Kwang, Warin Chamrap District, Ubon Ratchathani Province, Thailand). A Magnum Electric Motor machine with a 70 HP power of Kubota M7040, chopped the cassava tops into 2-cm-long pieces and sun-dried them at ambient temperature for 3 days. Dried cassava tops were replaced with dried cassava pulp at a ratio of 15:85 (gm/gm) on a DM basis in a tank with dimensions of 100 L for 21 days. Strain CNCM-1070, Levucell SC20 (r) SC, and its ingredient, 1010 CFU/gm of *S*. *cerevisiae,* were bought from Ubon Ratchathani Province, Thailand. 20 gm of *S*.* cerevisiae* was used to prepare solution A. The yeast was aerobically stimulated with 40 gm of sugar dissolved in tap water in 660 ml, with oxygen flushed through the mixture for 30 min before fermentation. Additionally, 3 gm of urea was mixed with 50 ml of molasses in 830 ml of tap water to prepare solution B. A and B were mixed at a 1:1 (v/v) ratio, and then air-flushed for 1 h to obtain solution C. Finally, solution C was mixed with dried CtFCp at a 1:1 (v/w) ratio [2].

### Dietary treatment and experimental design

The study was conducted at the Experimental Farm of the Faculty of Agriculture, UBU of Thailand. Cows were carefully protected from humans, animals, and environmental conditions to minimize errors. Twelve females of Thai native cattle in the growing stage, 75% Thai native × 25% Lowline Angus cattle at 14 months of age with an initial weight of 97 ± 18.10 kg, were administered in a completely randomized design (CRD). The study consisted of three dietary treatments, each with four replications, and one animal was used per replication. The treatments consisted of dried CtFCp in concentrations of 67% and 33% (Table 1). Three different dietary treatments were implemented; each replicated four times with one animal assigned per replication. The dietary treatments consisted of 100% concentrate (Control; CON), CtFCp-33, and CtFCp-67, all based on DM. The concentration and CtFCp of chemical compositions are presented in Table 1. The CTFCp mixture was thoroughly mixed using an SM-3.0CR machine with the following specifications: 3 HP, 50 Hz, 220 volts, 20.0 amps, and 1450 r/min. It was then stored in a 100 L plastic tank, located in the study area. Feed was provided twice daily at 7:00 am and 4:00 pm at 1% DM of body weight. In addition, clean water and a mineral salt block were offered ad libitum. Rice straw (RS) was fed to all cows ad libitum daily, with 100 gm/kg DM refusal of the RS offered. A bucket size of 40 × 60 cm was used for RS and 30 × 40 cm for mixed feed. All cows were placed in individual pens measuring 2.5 × 4 m, which are equipped with a concrete floor and iron walls. Before the trial, cows were injected with vitamin ADE at 1 ml/50 kg BW and 1% w/v of Ivermectin at 1 ml/50 kg BW.

**Table 1.** chemical composition of the concentration, dried CtFCp, and RS.

Items	Concentrate	CtFCp	RS
Ingredients, gm/kg DM			
CSP	-	764	-
Dry cassava leaves	-	150	-
Cassava chip	410	-	-
Soybean meal	140	-	-
Palm kernel meal	90	-	-
Corn meal	135	-	-
S. cerevisiae	-	2	-
Rice brand	130	-	-
Urea	20	30	-
Molasses	50	50	-
Sugar	-	4	-
Salt	5	-	-
Sulfur	10	-	-
Monodicalcium phosphate (P ≥ 21٪, Ca ≥ 14٪)	5	-	-
Mineral premix	5	-	-
Chemical composition, gm/kg DM			
DM	960.4	381.0	854.2
Ash	59.7	72.1	104.3
OM	940.3	927.8	895.7
CP	150.9	102.6	40.6
NDF	285.2	492.6	723.4
ADF	184.7	372.1	577.7
Ether extract	45.7	11.3	14.0
Acid insoluble ash	22.9	55.3	53.1

Note: P = Phosphorus; Ca = Calcium, DM = dry matter.

### Feed intake

All cows were adapted for 10 days before the experiment began to familiarize them with the palatability of the feed, their living environment, the flavor, and the feed provider. During this period, they were housed in individual pens. All cows were initially weighed to adjust DM feed intake. The experiment was conducted over 21 days, with 14 days dedicated to assessing feed intake, and samples were collected during the last 7 days. Daily, in the morning, the feed consumed and refused were recorded before new feed was provided.

### Sample collection and measurement

The refused feeds, including RS, concentrate, and a mixture of dried CtFCp, were recorded daily during the last 7 days of the 21-day experiment. All the samples were taken and dried in a hot air oven at 70°C, and then stored in their dry form for chemical analysis on a weekly basis. Before the feed was supplied, feces were collected individually from the rectum at 7:00 am daily during the last 7 days. The collected feces were mixed and stored at −20°C for subsequent nutrient and chemical analysis. The frozen fecal samples were thawed and dried in a hot air oven at 70°C for 72 h for chemical composition analysis. All the samples were ground through a 0.5 mm screen (Tecator, Hoganas, Sweden) and analyzed for DM, ash, and CP [20] with a NDF and ADF [21]. The apparent nutrient digestibility was estimated using acid-insoluble ash (AIA) as an indicator [16]. The rumen fluid and blood samples were collected at 0 and 4 h post-feeding on the last day of the experiment. On the day of the experiment, blood samples and rumen fluid were collected at 0 and 4 h post-feeding. A total of 10 ml of blood was collected from the jugular vein and separated into two parts, with 4 ml placed in a container with a serum cloth activator for blood urea nitrogen (BUN), triglyceride, creatinine, and total protein analysis.

Another 4 ml for glucose analysis was placed in a test tube containing sodium fluoride/EDTA. All chemical composition analyses were conducted according to the methods described by Küme et al. [22] and Zhang et al. [23]. A vacuum pump was used to collect rumen fluid by attaching a stomach tube; approximately 100 ml of rumen fluid was collected. The pH of rumen fluid was measured immediately after collection by using a glass electrode pH meter of the HANNA instrument 8,424 microcomputer, Singapore. The rumen fluid was filtered with a four-layer cheesecloth, and 1 M of sulfuric acid (H₂SO₄) was used at a 1:9 ratio for chemical analysis. The solution of rumen fluid was stored at −20°C. The solution of rumen fluid was concentrifuged at 3,500 rpm for 15 min to obtain the supernatant for volatile fatty acid analysis (VFA). The supernatant of the rumen fluid was filtered using a 0.45 μm Millipore filter before being injected into the chromatographic apparatus. Sulfuric acid (H₂SO₄) (0.005 mol/l) was used as the mobile phase in VFA analyses using a Dionex UHPLC Thermo Scientific UltiMate 3,000 linked to a C18 (4.6 × 250 mm) column (Chromeleon Dionex Corp), with UV-Vis detection at 210 nm [24]. VFA was used to estimate methane (CH₄), as described by Moss et al. [25]. The predicted methane (CH₄) concentration can be calculated using the formula: methane (CH₄) = (0.45 × acetic acid) − (0.275 × propionic acid) + (0.40 × butyric acid). A 10% solution of formalin was added to 9 ml of rumen fluid and kept in a refrigerator for protozoa and fungal zoospore counting by using a hemocytometer under a microscope [26]. According to Robinson et al. [27], the equation was used to calculate ME. The digestible nutrient of organic matter fermented in the rumen (DOMR) was calculated using the equation described by Lai et al. [3], as follows:

DOMR, kg/day = digestible organic matter intake (DOMI, kg/day) × 0.65.

Where,

DOMI = [digestibility of organic matter, kg/kg DM × organic matter intake, kg/day] /100.

The following conversion factors were applied: 1 kg DOMI = 15.9 MJ; ME/kg microbial CP (MCP kg/day) = 0.00825 × ME intake (MJ/day) [26].

#### Statistical analysis

Data were analyzed using the Statistical Package for the Social Sciences (version 16.0). The experiment followed a CRD with three dietary treatments and four replications. The model was used:


$$Y_{\mathrm{ij}}=\mu +T_{i}+{∊}_{\mathrm{ij}}$$

where *Y*_*ij*_ is the observation on the *j*-th animal receiving the -th treatment, *µ* is the overall mean, *T*_*i*_ is the fixed effect of treatment (CON, CtFCp-33, CtFCp-67), and is the random error term. For feed intake data recorded weekly, repeated measures analysis was conducted using the model:


$$Y_{\mathrm{ij}}=\mu +T_{i}+W_{j}+(T\times W{)}_{\mathrm{ij}}+\mathrm{AK}(T_{i})+{∊}_{\mathrm{ijk}}$$

where *W*_*j*_ is the effect of week, is the treatment × week interaction, represents the random effect of the animal within treatment, accounting for repeated observations over time. Treatment means were compared using Duncan’s Multiple Range Test. Statistical significance was declared at *p* < 0.05, and trends were discussed when 0.05 < *p* < 0.10. Results are expressed as the mean ± standard error of the mean (SEM).

## Results

### Chemical profile of concentrate, CtFCp, and RS feeds

Table 1 presents the chemical composition of concentrate, dried CtFCp, and RS. The current research revealed that CP of concentrate was 150.9 gm/kg DM, whereas that of the CtFCp was 102.6 gm/kg DM. In contrast to the concentrated diet, the CtFCp diets showed increased NDF and ADF levels, rising by 207.4 and 187.4 gm/kg of DM, respectively. This increase is attributed to the inherently higher fiber content of CtFCp compared to the concentrate. Nevertheless, RS contained 40.6 gm/kg of CP was lower than that of concentrate and CtFCp. However, NDF and ADF contents were substantially higher, which likely contributed to reduced digestibility.

### Feed intake characteristics, estimated energy, and MCP

DM intake, expressed in gm/kg BW0.75, demonstrated a significant decrease (*p* < 0.05), with the CON group exhibiting higher feed intake compared to the CtFCp-33 and CtFCp-67 groups. The estimated ME intake, expressed in MJ/kg DM, revealed a significant difference (*p* < 0.05), with the CON and CtFCp-33 groups exhibiting higher energy intake than the CtFCp-67 group. MCP differed in consistency compared to the group and showed a significantly positive statistic (*p* < 0.05). Despite having the lowest ME intake, the group CtFCp-67 still exhibited a positive and significant MCP output. The lower energy intake, possibly due to a different fermentable profile or other influencing factors, may have led to a more efficient conversion of available energy and nitrogen into MCP. In contrast, the CtFCp-33 and CON groups had higher ME intake, which is generally associated with increased MCP production, reinforcing the fundamental link between energy supply and microbial growth (Table 2).

**Table 2.** Feed intake and feed utilization of cattle fed different concentrations of dried CtFCp.

Variable	CON	CtFCp-33	CtFCp-67	SEM	*p*-value*
DM intake concentrate; concentrate with dry FCSP					
kg/day	1.18	1.45	1.16	0.07	0.150
٪BW	1.06^c^	1.49^a^	1.30^b^	0.05	< 0.001
gm/kg BW0.75	35.35^c^	47.94^a^	39.94^b^	1.65	< 0.001
RS					
kg/day	2.01^a^	0.80^b^	0.79^b^	0.18	<0.001
٪BW	1.85^a^	0.80^b^	0.79^b^	0.16	<0.001
gm/kg BW0.75	61.29^a^	27.26^b^	26.49^b^	5.20	<0.001
Total intake					
kg/day	3.19^a^	2.33^b^	2.12^b^	0.18	0.003
٪BW	2.91^a^	2.34^b^	2.15^b^	0.12	0.005
gm/kg BW0.75	93.27^a^	75.19^b^	68.31^b^	5.60	0.001
Estimate energy intake					
ME, MJ/kgDM	3.37^a^	2.63^a^	1.52^b^	0.29	0.005
ME, MJ/kgDM/day	53.59^a^	41.81^a^	24.28^b^	4.63	0.005
ME, MJ, gm/kgBW0.75	101.72^a^	92.13^a^	51.34^b^	8.94	0.010
Estimate microbe					
MCP, gm/kg/day	419.35^a^	319.01^bc^	221.92^bc^	37.59	0.040

Note:^a–c^ Values on the same row with different superscripts differ (*p* < 0.05). 100% concentrate (CON), 67% concentrate + 33% CtFCp (CtFCp-33), and 33% concentrate + 67% CtFCp (CtFCp-67); S.E.M = standard error of the mean.

### Nutrient intake and digestion

Table 3 shows that OM digestibility was not significantly different among the groups CON, CtFCp-33, and CtFCp-67 (*p* > 0.05). However, the digestibility of CP of CtFCp-33 and CtFCp-67 was significantly higher than that of the CON group (*p* < 0.001). The digestibility of NDF in the CON group was significantly higher than in the other groups, followed by CtFCp-67, which was digested more effectively than CtFCp-33. The percentage of ADF digestibility and nutrient intake of OM were not statistically different among the groups (*p* > 0.05). However, DM, CP, EE, NDF, and ADF intake differed significantly among groups (*p* < 0.05). Superscripts within the same row indicated no significant difference between the CON and CtFCp-33 groups, whereas the CtFCp-67 group differed significantly from both.

**Table 3:** Nutrient intake and digestibility of cattle fed different concentrations of dried CtFCp.

Variable	CON	CtFCp-33	CtFCp-67	S.E.M.	p-value*
Nutrient intake, kg/h/day					
DM	4.82a	3.60ab	2.73b	0.33	0.020
OM	4.96	4.48	3.63	0.27	0.110
CP	0.46a	0.40ab	0.30b	0.07	0.040
Ether extract	0.15a	0.10ab	0.08b	0.01	0.004
NDF	3.71a	2.92ab	2.34b	0.23	0.030
ADF	2.34a	1.89ab	1.59b	0.13	0.040
Nutrient digestibility, ٪					
DM	59.64a	49.54b	49.43b	1.59	< 0.001
OM	65.63	65.15	65.18	0.37	0.570
CP	75.08a	58.91b	56.49b	2.60	< 0.001
Ether extract	86.58a	76.50b	73.04c	1.78	< 0.001
NDF	62.88a	61.43a	58.70b	0.66	0.010
ADF	39.63	37.79	38.01	0.80	0.640
Total digestible nutrients	69.17a	66.14ab	66.01b	0.48	< 0.001

Note: a–c Values on the same row with different superscripts differ (p < 0.05). 100% concentrate (CON), 67% concentrate + 33% CtFCp (CtFCp-33), and 33% concentrate + 67% CtFCp (CtFCp-67); S.E.M.=standard error of the mean.

### The blood metabolites, temperature, and rumen pH

Table 4 shows rectal temperature, protozoal and fungal populations, rumen potential hydrogen, and glucose, and demonstrates an insignificant difference between 0 and 4 h post-feeding (*p* > 0.05) between the CON, the CtFCp-33, and the CtFCp-67 groups. The group CON had more BUN than groups CtFCp-33 or CtFCp-67, but the difference was insignificant after 4 h. Whereas creatinine, triglyceride, and total protein serum levels were insignificant at 0 and 4 h post-feeding in the CON, CtFCp-33, and CtFCp-67 groups (*p* > 0.05).

**Table 4.** Rectal temperature, rumen variables and blood metabolites of cattle fed different concentrations of dried CtFCp.

Variable	CON	CtFCp-33	CtFCp-67	S.E.M.	*p*-value*
Rectal temperature, °C					
0 h post-feeding	38.60	38.55	38.78	0.09	0.590
4 h post-feeding	39.52	39.70	39.12	0.13	0.170
Protozoa log cell/ml					
0 h post-feeding	4.32	4.41	4.22	0.06	0.460
4 h post-feeding	4.26	4.30	4.14	0.05	0.460
Fungi log cell/ml					
0 h post-feeding	3.91	3.93	3.77	0.03	0.460
4 h post-feeding	3.81	3.71	3.61	0.17	0.250
Rumen pH					
0 h post-feeding	6.44	6.27	6.18	0.10	0.630
4 h post-feeding	6.16	6.00	6.31	0.07	0.150
Glucose, mg/dl					
0 h post-feeding	77.25	71.50	61.00	3.64	0.190
4 h post-feeding	70.75	67.75	64.00	1.26	0.070
BUN, mg/dl					
0 h post-feeding	12.25ab	13.75a	12.75ab	2.84	0.020
4 h post-feeding	15.50	15.02	15.00	0.38	0.600
Creatinine, mg/dl					
0 h post-feeding	1.28	1.21	1.17	0.04	0.660
4 h post-feeding	1.34	1.29	1.15	0.04	0.190
Triglyceride, mg/dl					
0 h post-feeding	35.00	38.00	40.75	2.90	0.760
4 h post-feeding	35.75	45.25	50.00	3.21	0.190
Total protein-serum, gm/dl					
0 h post-feeding	6.98	6.85	6.45	0.23	0.670
4 h post-feeding	6.88	6.60	6.32	0.15	0.360

Note: a,b Values on the same row with different superscripts differ (p < 0.05). 100% concentrate (CON), 67% concentrate + 33% CtFCp (CtFCp-33), and 33% concentrate + 67% CtFCp (CtFCp-67); S.E.M. = standard error of the mean; 0 h = 0 hours; 4 h = 4 hours; mg/dl = milligram/deciliter; gm/dl = gram/deciliter.

### Rumen VFA production

The rumen VFA, including total volatile fatty acid (TVFA), acetic acid (C2), propionic acid (C3), and butyric acid (C4), is shown in Table 5. From 0 and 4 h, the average TVFA levels varied from 94.70 to 100.45 and 103.70 to 111.15 mmol/l, respectively, with no statistically significant difference between the groups (*p* > 0.05). At 4 h post-feeding, acetic acid (C2) altered differently between groups (*p* < 0.05). The usage for CtFCp-33 was greater than that of the CON or CtFCp-67 groups. Propionic acid (C3) did not affect any of the groups at 0 h (*p* > 0.05). Still, it did have an effect at 4 h (*p* < 0.05), with the CON and CtFCp-33 groups exhibiting the greatest effect compared to the CtFCp-67 group. Butyric acid (C4) did not differ between groups (*p* > 0.05) at 0 h but changed at 4 h (*p* < 0.05). The group usage of CtFCp-67 was the lowest compared to any other groups, with the CON and CtFCp-33 groups in the same row superscript. Nevertheless, the ratio of (C2:C3) showed a significant difference both at 0 and 4 h (*p* < 0.05). The group using CtFCp-33 had the most favorable outcome, while the other two groups showed similar results. Methane (CH₄) emissions estimated for the CtFCp-33 group were positively higher than the CON and CtFCp-67 groups (*p* < 0.05), as shown in Table 5.

**Table 5.** Ruminal fermentation and VFA concentrations of cattle fed different concentrations of dried CtFCp.

Variable	CON	CtFCp-33	CtFCp-67	S.E.M.	*p*-value*
TVFA, mmol/l					
0 h post-feeding	111.15	100.45	109.95	3.94	0.520
4 h post-feeding	94.70b	103.70ab	110.00a	3.06	0.020
VFA profiles, mmol/100 mol					
Acetic acid (C2)					
0 h post-feeding	56.74	63.89	61.59	1.37	0.080
4 h post-feeding	63.20b	68.44a	61.98b	1.02	0.005
Propionic acid (C3)					
0 h post-feeding	35.92	28.64	31.60	1.27	0.050
4 h post-feeding	32.33a	26.23b	30.77a	0.93	0.003
Butyric acid (C4)					
0 h post-feeding	7.47	7.47	6.80	0.32	0.660
4 h post-feeding	4.36b	5.33ab	5.86a	0.31	0.100
Acetic: Propionic acid ratio (C2: C3)					
0 h post-feeding	1.60	2.27	1.98	0.12	0.060
4 h post-feeding	1.97b	2.61a	2.02b	0.10	0.002
Estimated methane (CH4), gm/day					
0 h post-feeding	18.67b	23.86a	21.74ab	0.92	0.040
4 h post-feeding	21.23b	25.72a	22.33b	0.67	0.003

Note: ^a,b^ Values on the same row with different superscripts differ (p < 0.05). 100% concentrate (CON), 67% concentrate + 33% CtFCp (CtFCp-33), and 33% concentrate + 67% CtFCp (CtFCp-67); significantly (p < 0.05); S.E.M. = standard error of the mean.

## Discussion

The CP content in the concentrate wassimilar to that reported by So et al. [28], but less than that reported by Goiri et al. [29], and higher than that reported by Sommai et al.[30]. However, the CP in fermented cassava pulp (FCSP) was lower than that reported by Sommai et al.[30]. Previous research aligns with these findings regarding CP content in both concentrate and fermented cassava [2,30]. For example, yeast-fermented CtFCp showed a protein content of 15.05%, while CSP protein content increased to approximately 17% [17]. The altered CP content may be influenced by varying the levels of urea and yeast during fermentation. The CP content increased from 2.6% to 11.05% of CSP while adding *S*. *cerevisiae* and look-pang mixture starter structure. Fermentation of yeast to CtFCp enhances nutrient availability, improves digestibility and nutritional availability, and can substitute grain in rumen feed ingredients [2]. These results also supported [2,12,31]. Therefore, CtFCp is crucial to emphasize the need for alternative feed resources, particularly in terms of energy gain requirements. In addition, the findings underscore the potential for CtFCp to support sustainable livestock systems by reducing feed costs and minimizing agricultural waste [2].

Sommai et al. [30] demonstrated that replacing soybean meal with FCSP with yeast enhanced nutrient digestibility, including OM and CP. Similarly, Khejornsart et al. [17]demonstrated that FCSP mixed as total rations for lambs enhanced nutrient digestibility, including DM, OM, CP, and NDF. Juckem et al. [32] demonstrated that high-concentrate diets improve the digestibility of OM and enhance milk yield in goats. In dairy cattle, increasing dietary starch levels can reduce methane (CH₄) production per unit of estimated rumen-fermentable OM. Conversely, diets with excessive fiber may fail to meet the energy requirements of feedlot cattle, particularly for fattening and the synthesis of intramuscular fat, which is closely associated with marbling scores [33]. Furthermore, the current study was supported by Lai et al. [3], who reported that a 70% concentrate-to-forage ratio in yak diets increased DM intake and nutrient digestibility. Similarly, Gunun et al. [34] found that replacing more than 50% of the feed concentrate with yeast-fermented cassava peels or cassava peels fermented with effective microorganisms has no effect on feed intake, nutrient digestibility, and rumen fermentation.

This substitution may also decrease feed expenses by as much as 32% for each unit of weight gain. These results further confirm the potential benefits of incorporating fermented cassava by-products into ruminant diets [11,30,35].

High-concentration diets in cows demonstrated significantly increased nutrient digestibility and intake, but this finding differed from some earlier studies [36]. A roughage and concentrate mixed in a ratio of (30:70) can improve the population of bacteria and nutrient digestibility, as it contains more available energy. This could be because the concentrate may provide additional nutrients through enhanced rumen fermentation and rumen microbial growth [11]. In addition, using high-concentrate feed, a reduced ruminal ammonia concentration, and expanded ammonia utilization proved to be good conditions for microorganisms to digest feed [18]. Furthermore, the activation of β-glycosidase hydrolase enzymes and polysaccharides in yeast can enhance digestive function in ruminants. Additionally, urea in the fermentation solution may contribute to the breakdown of the fiber structure in CSP, acting as an alkaline agent (ammonium hydroxide) [37]. The diminished digestibility of ADF and NDF may be ascribed to a propensity for reduced ruminal pH, potentially induced by elevated concentrations of soluble carbohydrates in the diet. This current study has prior support from Khejornsart et al. [17], who demonstrated thatNDF was well digested in sheep fed a total ration with added CSP over a 21-day experiment. In contrast, [36] found that the CP digested was at a constantly low level in cattle fed a CSP diet.

### Blood metabolites, temperature, rumen pH, and microbial population

The dietary treatments did not affect rectal temperature, protozoal and fungal populations, ruminal pH, or BUN, according to recent results. However, some studies [2,11,30] have reported conflicting findings. In this study, increasing the inclusion of CtFCp up to 67% did not affect ruminal pH, which remained within the optimal physiological range. This stability may be attributed to the CtFCp fermentation process, which produces a product with a beneficial level of live yeast, primarily *S*. *cerevisiae*, known for its role in maintaining rumen health. It has been demonstrated that yeast plays a crucial role in preserving a healthy ruminal environment by promoting the growth of lactate-utilizing bacteria such as *Megasphaera elsdenii*. Increasing lactic-acid-utilizing bacteria in the rumen inhibits the bacteria produced by lactate activity, reduces lactic acid accumulation, and maintains a stable rumen pH [38]. The yeast supplementation in dietary concentrate could be considered due to its impact on the rumen pH effect [7].

Nevertheless, yeast fermentation helps prevent ruminal acidosis, a serious condition that impairs digestion and negatively affects overall animal health. Therefore, the current study found that pH, as agreed [39], ranged from 6.67 to 6.75, which supports microbial digestion. Glucose, creatinine, triglyceride, and total protein levels in the serum didn’t alter at both 0 and 4 h post-feeding among the CON, CtFCp-33, and CtFCp-67 groups. TVFA, C2, C3, and C4 levels were unaffected at 0 h post-feeding; however, C2 and C3 levels increased after 4 h, as reported by some [19,36]. In contrast, others, such as So et al. [28], disagree. Some reports have revealed that high concentrations supplied in diets enhanced C3, and a comparison of concentrate proportioned with a high-forage diet found that TVFA and molar proportions of C2 were negatively altered [40]. The current study results employed CtFCp-67 and CtFCp-33, and C2 and C4 increased. This finding was supported h research from Jiang et al. [8], who found that yaks were fed a high concentrate with a high forage diet 4 h post-feeding. Thus, rumen fermentation alters structural carbohydrates into a non-structural form. As a result, the high concentration used may affect rumen microbes. Therefore, C4 production increased due to the low ruminal pH, which stimulated the growth of certain ruminal microbes, including Succiniclasticum, Ruminococcus, Butyrivibrio, and Mogibacterium, in the rumen epithelium [41].

## Conclusion

Feeding dried CtFCp-33 improved nutrient intake and digestibility more effectively than CtFCp-67 in Thai native cattle. Diets in the CtFCp-33 and CON groups also resulted in higher TVFA concentrations and butyrate production. These findings indicate that CtFCp is a valuable alternative feed resource for ruminants. Specifically, it can replace up to 33% of concentrate in the diet of growing Thai native cattle without negatively affecting feed intake, nutrient digestibility, or digestive health. Future research should investigate the effects of CtFCp-based diets on lactating cows and the quality of meat produced.

## List of abbreviations

C2, acetic acid; C3, propionic acid; C4, butyric acid; CR, cassava root; CSP, cassava pulp; CtFCp, dried cassava tops fermented cassava pulp; DOM, digestibility of organic matter; DOMI, digestible organic matter intake; DOMR, digestible nutrient of organic matter fermented in the rumen; FCSP, fermented cassava pulp; IBW, initial body weight; MCP, microbial crude protein; RS, rice straw; TVFA, total volatile fatty acid; VFA, volatile fatty acid(s).
